# The Effects of Age and Treatment Intensity on Behavioral Target Mastery With Applied Behavior Analysis (ABA) Intervention Using Causal Moderation Models

**DOI:** 10.7759/cureus.67179

**Published:** 2024-08-19

**Authors:** Tami Peterson, Jessica Dodson, Robert Sherwin, Frederick Strale

**Affiliations:** 1 Hyperbaric Oxygen Therapy, The Oxford Center, Brighton, USA; 2 Applied Behavior Analysis, The Oxford Center, Brighton, USA; 3 Hyperbaric Oxygen Therapy, Wayne State University School of Medicine, Detroit, USA; 4 Biostatistics, The Oxford Center, Brighton, USA

**Keywords:** applied behavioral analysis (aba), interaction effects, causal moderation analysis, aba treatment effects, age group differences, aba efficacy, age groups

## Abstract

Introduction

Applied behavior analysis (ABA) is a therapy that focuses on improving specific behaviors using positive and negative reinforcement through antecedents, behaviors, and consequences, particularly in individuals with autism and other developmental disorders. It uses the principles of learning theory to bring about meaningful and positive changes in behavior. In ABA treatment, intensity refers to the amount and frequency of therapy an individual receives. This includes weekly hours, session trials, and overall duration. Intensive treatment involves more hours and trials tailored to individual needs and responses. Younger individuals, particularly those with autism, often receive more intensive therapy because early intervention leads to better outcomes. Programs may recommend 25-40 hours per week for young children. As children age, therapy may become less intensive, focusing on specific skills. The study explores how age and treatment intensity affect the mastery of behavioral targets in ABA interventions.

Materials and methods

This study involved 100 participants (89 children, four adults, and seven instances where the individuals' ages were not recorded due to random data entry errors (MCAR)) who received ABA treatment over three months. The treatments included functional analysis, discrete trials, and mass and naturalistic training. Data on the mastery of target behaviors were collected using the Catalyst software (New York, New York). The primary outcome was the percentage of mastered behavioral targets, indicating the effectiveness of the ABA treatment. Several predictors were examined, including the participant's age and treatment intensity variables, such as the average number of trials and teaching days to achieve behavioral mastery. The interaction effects between age and these treatment intensity variables were analyzed. The study used descriptive and inferential statistics to explore these interactions, including correlational and multiple regression analyses with causal moderator modeling.

Results

In Model 1, a baseline multiple regression analysis showed that average teaching days significantly predict the percentage of targets mastered. However, its limited explanatory power suggests other variables also play a role. Model 2 introduced interaction effects using causal models, revealing that age moderates the relationship between treatment variables and behavioral outcomes. This model provided a more nuanced understanding but still had room for improvement. Model 3 further refined the approach, achieving higher R-values and lower standard error. It highlighted age's significant role in modifying the impact of teaching days on mastery. This model's superior performance emphasizes the importance of considering age as a moderating factor in ABA interventions, leading to more effective and personalized behavior therapy.

Conclusions

This study significantly enhances our understanding of the complex interactions between age and treatment intensity within ABA interventions. Practitioners and researchers can develop more tailored and effective therapeutic strategies by identifying and leveraging these interactions. This approach optimizes the treatment process and ensures that interventions are personalized to meet the unique needs of each individual. Ultimately, this leads to more successful outcomes in behavioral therapy, fostering improved adaptive behaviors and overall development.

## Introduction

The prevalence of autism spectrum disorder (ASD) is estimated to be approximately one in every 36 children, per the Centers for Disease Control and Prevention (CDC). This statistic underscores the widespread nature of ASD, which is a pervasive neurodevelopmental disorder. It is important to note that ASD transcends the boundaries of ethnicity, race, and socioeconomic status. The incidence of ASD is observed ubiquitously across all these demographic groups, indicating that the disorder is not confined to any specific population segment. This universal prevalence underscores the necessity for inclusive and comprehensive strategies in ASD diagnosis, treatment, and support services [1,2].

ASD is a complex neurodevelopmental condition that manifests through persistent impairments in social communication and interpersonal interactions across various contexts. A triad of core symptoms typically characterizes this disorder. Individuals with ASD often exhibit a marked deficit in social-emotional reciprocity. This can range from an abnormal social approach and failure in normal back-and-forth conversation to reduced sharing of interests, emotions, or affect and response to social interactions [3]. ASD is associated with challenges in using nonverbal communicative behaviors for social interaction. This includes abnormalities in eye contact and body language or deficits in understanding and using nonverbal communication, all of which are essential for social interaction. Individuals with ASD may demonstrate insufficiencies in the development, maintenance, and knowledge of relationships. This can manifest as difficulties adjusting behavior to suit various social contexts, difficulties in sharing imaginative play and making friends, or an apparent absence of interest in people. ASD is a multifaceted neurodevelopmental disorder that affects social communication and interaction, with symptoms varying significantly among individuals [3].

Applied behavior analysis (ABA) therapy is widely regarded as the gold standard for treating ASD, supported by extensive research and substantial evidence [4-7]. Other effective therapies include developmental approaches such as speech and language, occupational therapies such as sensory integration, physical therapies, and educational, social-relational, pharmacological, psychological (cognitive-behavioral), and complementary/alternative treatments [8].

A meta-analysis of 14 randomized controlled trials with 555 participants demonstrated that ABA has a moderate to high effect in treating ASD [9]. Additionally, a systematic review of 29 studies found ABA programs to be mild to highly effective, offering significant benefits to children with ASD [2,10]. In a randomized controlled trial involving 28 children with autism, the most significant improvements in intelligence scores were observed in those receiving comprehensive ABA therapy [2,11]. Despite its proven effectiveness, ABA adoption remains limited due to misunderstandings, misconceptions, and challenges in determining appropriate research methods to evaluate individualized interventions. The impact of ABA on autism symptom severity, language development, and school placement remains uncertain due to insufficient data and a lack of follow-up studies [2,12].

ABA interventions have shown moderate effects on intellectual functioning and adaptive behaviors, but they did not surpass control groups in improving language abilities, symptom severity, or parental stress [2,13]. A thorough review identified studies examining ABA's impact on health outcomes, categorizing these outcomes into eight areas: cognitive, language, social/communication, problem behavior, adaptive behavior, emotional, autism symptoms, and quality of life [2,14]. Despite strong evidence supporting ABA, researchers have highlighted an "efficacy-effectiveness gap" due to individual differences, lower compliance rates, less standardized treatments, and financial constraints [2,15]. ABA remains a popular and widely preferred treatment method, with therapy rankings varying based on each child's unique needs. Other therapies include physical, speech, nutritional, occupational, and cognitive-behavioral therapy, play therapy, social skills training, and developmental therapies [2,16,17].

In the context of ABA, "treatment intensity" refers to the amount and frequency of therapeutic intervention provided to an individual. It encompasses the number of hours per week of therapy, the number of trials or teaching attempts made during each therapy session, and the duration over which the therapy is provided. High-intensity treatment typically involves more hours of therapy per week, more trials per session, and a longer duration of therapy. Treatment intensity can be adjusted based on individual needs and responses to the therapy. It is crucial to acknowledge that the intensity of the treatment can profoundly influence the outcomes of ABA therapy. Children who receive intensive treatment have been observed to make clinically significant gains [2,14-22].

Virues-Ortega et al. discovered that increased intervention time, lower age at intervention onset, and higher pre-intervention functioning might be associated with greater intensive behavior intervention (IBI) outcomes for intervention programs of up to four years in duration. Their study provided the methodological basis for predictor identification in the longitudinal analysis of IBI [16].

Eldevik et al. reported their results from regression analyses, showing that high intervention intensity was the only variable that independently predicted intelligence quotient (IQ) and adaptive behavior composite (ABC) gain. In both cases, high intensity (36+ weekly intervention hours) was associated with larger gains. In addition, ABC scores at intake and IQ scores at intake predicted gains in ABC [17].

Linstead and colleagues have expressed the need for additional research to understand the impact of the duration of treatment. They referenced the work of Granpeesheh et al., who proposed that using achieved learning objectives as an outcome variable in quasi-experiments could enable tracking short-term results, which standard diagnostic assessment scales are not equipped to do [15,18]. Using achieved learning objectives as the outcome could be advantageous as it offers a broad and socially meaningful evaluation of treatment progress. They underscored the importance of assessments specific to treatment that focus on achieved learning objectives to gauge short-term successes or failures. They highlighted this as a significant dependent measure and pointed out the role of the duration and intensity of treatment as a crucial determinant of the mastery of target behaviors [15,18].

Reickow and Wolery, in their systematic review and meta-analysis, found that early intensive behavioral interventions were, on average, an effective treatment for children with autism. However, the authors cautioned in interpreting the results due to gaps and limitations in the evidence base. They commented that the heterogeneity found in their meta-analysis was further explored with moderator variables and that such analysis of moderator variables suggested pointers for further research [20].

In their systematic review and meta-analysis on the efficacy of ABA treatment, Eckes et al. found that many studies show promising results for ABA-based interventions in treating ASD. They noted that to thoroughly determine the validity of comprehensive ABA-based interventions for ASD, more methodologically sound studies are necessary [13]. Consequently, robust conclusions on effectiveness are limited by the small number of primary studies and their low quality. This limitation also applies to the moderating influence of treatment modalities: treatment intensity and child characteristics, such as age. Knowledge regarding causal moderators would assist professionals in deciding on appropriate treatments, which would assist parents of ASD children in making informed decisions [12].

Studies focusing on moderating effects of treatments (such as intensity) and child variables (such as age) are crucial in predicting treatment outcomes. The intensity of treatment can significantly impact therapy effectiveness, with more intensive treatments potentially leading to greater improvements. However, optimal intensity may vary based on individual needs. Similarly, a child's age can influence treatment response due to developmental differences, necessitating age-appropriate strategies. By examining these factors, researchers can better tailor treatments to individual needs, potentially enhancing therapy effectiveness and efficiency, and ultimately improving the quality of life for those undergoing treatment.

In view of this, our study aims to investigate how age and treatment intensity interact and their moderating impacts on behavioral outcomes, specifically the percentage of behavioral targets mastered, as a result of ABA intervention.

## Materials and methods

Research participants and context

A total of 100 individuals (89 children, four adults, and seven instances where the individuals' ages were not recorded due to random data entry errors (MCAR)) underwent ABA treatment incorporating functional analysis, discrete trial training, mass trials, and naturalistic training. The study spanned three months, from March 19, 2023, to June 11, 2023 [5]. Data on the mastery of target behaviors were gathered retrospectively through a chart review within the "Catalyst" tracking software. All participants diagnosed with autism received treatment at The Oxford Centers (TOC), located in Brighton and Troy, Michigan, United States. This institution specializes in a mixed-methods approach to ABA. This approach includes discrete trial training, mass trials, and naturalistic environment training treatment modalities [22].

In advance of ABA training, one of the eight Board-Certified Behavioral Analysts (BCBAs) crafted a personalized treatment plan for each participant, considering the individual's unique needs and objectives. Over three months, each participant was assigned to one of the 83 behavioral technicians, potentially working with a team of three to five technicians. Suitable materials were chosen and arranged in designated rooms for discrete trial training and mass trials or in a naturalistic environment that encourages interaction and provides opportunities for participants to engage in functional and meaningful real-world scenarios. The assignment of behavioral technicians to individuals varies daily, with each participant receiving an average of four to seven hours of treatment per day, amounting to a minimum of 25 hours per week to a maximum of 40 hours a week [22].

Teams of behavioral technicians collected detailed data on specific behaviors and skills, focusing on the antecedent, the behavior itself, and the subsequent consequence. They tracked the individual's progress, gradually reducing prompts and reinforcements as the individual achieved mastery of the skills with an accuracy of 80%. They also monitored whether the individual could generalize and retain the skill. These data were inputted into a portable "Catalyst" database, which was then consolidated and updated daily into a central database [22].

Procedure for gathering data and defining variables operationally

Data on individuals diagnosed with autism who underwent ABA treatment were compiled through a retrospective chart review. This approach allowed for the comprehensive collection of relevant data over a specified period. This study's primary outcome variable, or the dependent variable, was the proportion of behavioral targets that the individuals could master over three months. This variable served as a quantifiable measure of the effectiveness of the ABA treatment.

Several predictor variables, or independent variables, were identified to potentially influence the outcome. These included the individuals' age (measured in years), the average number of trials required to achieve behavioral mastery (representing the intensity and duration of the treatment), and the average number of teaching days leading to behavioral mastery (also indicative of treatment intensity and duration).

These variables were carefully selected to provide a comprehensive understanding of the age and treatment intensity factors that could potentially influence the success of ABA treatment in mastering behavioral targets [22].

Outcome measure

Over three months, the quantification of behavioral targets accomplished served as a pivotal indicator, gauging an individual's advancement towards delineated learning goals and skill acquisition. The criterion for mastery, as established by the BCBA, was met when the individual demonstrated the ability to perform a specific task or skill with a precision rate of 80%. This benchmark underscores the rigorous standards set by the BCBA and provides a robust measure of an individual's progress and proficiency in the context of ABA [22].

Predictor variables

This study incorporated three predictor variables. "age" in years denotes the chronological age of the individuals, calculated based on a standard year. "Average trials to behavioral mastery," a metric indicative of the intensity and duration of treatment, signifies the count of behavioral responses necessitated to attain a predetermined level of behavioral performance (the mastery criterion) as established by the BCBA for a specific skill or behavior [22]. "Average teaching days to mastery’" is another measure of treatment intensity and duration, representing the number of days elapsed from the initial introduction of a target to its eventual mastery. The study participants underwent five full days of ABA treatment per week, with treatment protocols tailored to each individual's specific needs [22].

In addition to the three primary predictor variables, this study also incorporated two interaction variables, specifically (1) the interaction between "age" and "average trials to behavioral mastery," and (2) the interaction between "age" and "average teaching days to mastery." These interaction variables were created through a process known as "centering."

In the context of this study, "centering" involved converting the predictor variables into standardized z-scores. This was achieved by subtracting the mean of the variable from each score and then dividing the result by the standard deviation of the variable. The interaction standardized z-scores were then computed by multiplying the standardized z-scores of the two variables involved in each interaction. This method of creating interaction terms helps to reduce multicollinearity between the predictor variables and their interaction terms, thereby enhancing the interpretability and reliability of the regression coefficients [23].

The study utilized "Catalyst," a specialized ABA data collection software, to generate automated progress reports for outcome data pertaining to discrete trial teaching targets with frequency and rate data. The mastery criteria for target behaviors are defined in terms of the percentage of successful behavioral trials, the minimum number of behavioral trials, and the number of therapists achieving above an 80% criterion [24,25].

The Catalyst software is equipped with customizable graphs to monitor progress and/or lack thereof with targeted behaviors. It is designed to automatically identify mastered target behaviors as soon as the set criteria are met [24,25].

Data analysis

The entirety of the descriptive and inferential statistical analyses was conducted utilizing IBM SPSS Statistics for Windows, Version 29 (Released 2022; IBM Corp., Armonk, New York). For the inferential analysis, the nominal level of significance (α) was established at 0.05. Consequently, if the p-values are found to be less than 0.05 (p<0.05), the null hypothesis will be rejected, thereby inferring statistical significance [26].

The study will present an analysis of the descriptive demographics, inclusive of any missing values. Summary statistics will be generated for both categorical variables, such as gender and race/ethnicity, and continuous variables, including age, percentage of behavioral targets mastered, average trials to behavioral mastery, and average teaching days to behavioral mastery. These statistics will encompass measures of central tendency and dispersion, namely the mean and standard deviation, as well as the median and range.

A correlational analysis, employing Pearson r, was conducted on the variables: percentage of behavioral targets mastered, age, average trials to behavioral mastery, and average teaching days to behavioral mastery. This analysis aimed to discern any statistically significant or non-significant relationships between these variables.

A multiple linear regression analysis was conducted, with the percentage of behavioral targets mastered serving as the dependent (predicted) variable. The independent (predictor) variables included age, average trials to behavioral mastery, and average teaching days to behavioral mastery.

In addition, two interaction variables were considered: the interaction between "age" and "average trials to behavioral mastery," and the interaction between "age" and "average teaching days to mastery." These interaction variables were included to conduct a causal moderator analysis.

The causal moderator analysis is a statistical technique used to understand the conditions under which a particular effect occurs. In this context, it helps to understand how the age of the individual and the average trials or teaching days to behavioral mastery interact to influence the percentage of behavioral targets mastered. This analysis can provide valuable insights into the factors that moderate the effectiveness of the treatment, potentially leading to more tailored and effective interventions.

Institutional review board (IRB)

This investigation was executed retrospectively, utilizing data procured through a chart review conducted for clinical objectives. The study was subjected to a rigorous review process by the WIRB-Copernicus Group (WCG®IRB), subsequently receiving an exemption (approval number: 1-1703366-1). The authors solemnly affirm that the analytical procedures undertaken in this study were in strict compliance with the ethical guidelines delineated in the 1964 Declaration of Helsinki, along with its subsequent amendments or equivalent ethical standards. This adherence ensures the protection of the rights, safety, and well-being of the subjects involved in the study. It is noteworthy to mention that subsequent to the acquisition of the ClinicalTrials.gov Identifier NCT06043284, the Oxford Recovery Center (ORC) underwent a name change and is now recognized as The Oxford Center (TOC). Other identifiers associated with the study include OxRS-01-2021.

## Results

Descriptive statistical demographics

The study encompassed a sample of 100 individuals diagnosed with autism. The sample's age distribution was characterized by a mean of 8.88 ± 8.05 years, with a median age of seven years. The age range spanned from a minimum of one year to a maximum of 73 years, with seven instances where the age was not recorded. Regarding gender distribution, the sample comprised 74 males (74% of the n=100) and 25 females (25% of the n=100). There was one instance where the gender was not specified [26]. Concerning racial and ethnic composition, the sample included 72 individuals identifying as White (72% of the n=100), 12 as Asian (12% of the n=100), five as American Indian/Alaska Native (5% of the n=100), and four as Hispanic (4% of the n=100). Seven individuals did not specify their race or ethnicity (7% of the n=100). When categorized by age groups, 18 children (18% of the n=100) fell within the 1-4-year category, 39 children (39% of the n=100) were in the 5-8-year category, 20 children (20% of the n=100) were in the 9-12-year category, 12 children (12% of the n=100) were in the 13-16-year category, and four children (4% of the n=100) were in the 17-73-year category. There were seven instances where the age category was not specified. Four subjects were older than 17, specifically 18, 20, 25, and 73 [22].

No data were missing for critical metrics: average trials to mastery, average teaching days to mastery, and percentage of targets mastered. The mean for average trials to mastery was 118.18 ± 85.07 trials. The median was 108.36 trials. The range was 0 to 421.15 trials. The mean for average teaching days to mastery was 16.92 ± 10.11 days. The median was 16.11 days, ranging from 0 to 44.85 days. For the percentage of targets mastered, the mean was 55.33% ± 26.83%, and the median was 63.13%, ranging from 0% to 94.47%.

The data indicate a variation in the number of trials and days needed to reach mastery, a characteristic often observed in individuals with autism. The standard deviations point to a broad spread around the averages, a typical observation in autistic individuals. There was also a notable variation in the percentage of targets mastered, underscoring the differences in individual learning speeds or the complexity of the targets.

Inferential data analyses

A comprehensive multiple linear regression analysis was conducted on the outcome and predictor variables, thoroughly examining the underlying assumptions. Before implementing the multiple regression procedures, the study confirmed the independence of observations. This was evidenced by the fact that the measurements of each individual diagnosed with autism were distinct and uninfluenced by the measurements of other individuals in the sample. Each participant constituted a unique observation, and the value of one observation did not alter or impact the value of another, thereby eliminating any dependence [18]. The existence of a linear relationship between the dependent and independent variables was ascertained through the generation of bivariate scatterplots. Homoscedasticity, a critical assumption of linear regression, was validated as the standardized residuals were evenly distributed between -3 and +3 [22]. The study detected minimal multicollinearity, as evidenced by tolerance levels exceeding 0.10 (ranging from 0.305 to 0.971) and variance inflation factor (VIF) scores falling below 5 (ranging from 1.03 to 3.28). However, a significant correlation was identified in the correlation matrix between the variables "average trials to behavioral mastery" and "average teaching days to mastery," with a Pearson R-value of 0.826 and a p-value less than 0.001. The normality of residuals, another critical assumption of linear regression, was assessed via standardized residuals, which fell within an acceptable range (-1.724 to 1.968). Cook's distance identified one significant outlier in the dependent variable, specifically cases 9, 29, and 34 [22].

Correlation matrix for the outcome and predictor variables

As indicated in Table 1 below, a statistically significant correlation exists between average trials to mastery and average teaching days to mastery: Pearson correlation: 0.826, p<0.001 (interpretation: strong positive correlation; statistically significant). More trials are associated with more teaching days and average teaching days to mastery and % of targets mastered: Pearson correlation: 0.281, p=0.005 (interpretation: moderate positive correlation; statistically significant). More teaching days are associated with a higher percentage of targets mastered. These significant correlations suggest a strong relationship between the number of trials and teaching days and a moderate relationship between teaching days and targets mastered. Other correlations are not statistically significant. The correlation matrix for the outcome variable and the predictor variables are presented in Table 1 below.

**Table 1:** Correlation matrix for outcome and predictor variables **Correlation is significant at p<.05, 2-tailed The data have been represented as Pearson correlation, p-value, n, and CI CI: 95% confidence interval

Variable	Age	Average Trials to Mastery	Average Teaching Days to Mastery
Average trials to mastery			
Pearson correlation (CI)	-0.038 (-0.240, 0.167)		
p-value	0.716		
n	93		
Average teaching days to mastery			
Pearson correlation (CI)	0.034 (-0.171, 0.236)	.826** (0.752, 0.880)	
p-value	0.748	<0.001	
n	93	100	
% of targets mastered			
Pearson correlation (CI)	0.126 (0.080, 0.322)	0.142 (-0.056, 0.329)	.281** (0.089, 0.452)
p-value	0.228	0.159	0.005
n	93	100	100

Baseline multiple regression model for predictors on the outcome variable: Model 1

The multiple regression model summary indicates that for the dependent variable percentage of targets mastered and the predictor variables age, average trials to mastery, and average teaching days to mastery, R=0.355, R²=0.126, Adjusted R²=0.097, R² change=0.126, F-change=4.277, df=3,89, and p=0.007 (the model is statistically significant). The model explains 12.6% of the variance in the percentage of targets mastered.

As indicated in Table 2 below, the dependent variable is the percentage of targets mastered, while the predictor variables are age, average trials to mastery, and average teaching days to mastery. As for coefficients, the constant is B=38.568, the baseline value of the percentage of targets mastered when all predictors are zero. With age, the unstandardized coefficient (B) is 0.329, the standardized coefficient (β) is 0.100, with a t-value of 1.001, a p-value of 0.32, and a confidence interval (CI) of (-0.325, 0.984). Interpretationally, age has a small, non-significant positive effect on the percentage of targets mastered. With average trials to mastery, the unstandardized coefficient (B) is -0.076, the standardized coefficient (β) is -0.243, the t-value is -1.408, the p-value is 0.163, and the CI is (-0.183, 0.031). Interpretationally, there is a non-significant negative relationship with the percentage of targets mastered. With average teaching days to mastery, the unstandardized coefficient (B) is 1.322, the standardized coefficient (β) is 0.501, the t-value is 2.896, the p-value is 0.005, and the CI is (0.415, 2.229). Interpretationally, there is a significant positive relationship; more teaching days increase the percentage of targets mastered. Collinearity statistics in the form of tolerance and VIF (variance inflation factor) indicate that tolerance=0.986 and VIF=1.014 for age. Tolerance = 0.329 and VIF = 3.043 for average trials to mastery and average teaching days to mastery. These values suggest no serious multicollinearity issues. The model indicates a significant predictor, average teaching days to mastery, with non-significant predictors, age and average trials to mastery. The model suggests that increasing teaching days has a significant positive effect on mastering targets, while age and trials do not have a significant impact.

**Table 2:** Baseline multiple regression model for predictors on outcome variable: Model #1 **Correlation is significant at p<.05, 2-tailed The data are presented as unstandardized coefficients (B), standard error, standardized coefficients (β), t-values, p-values, 95% confidence interval for B (upper and lower bounds), and collinearity statistics (tolerance and VIF) Outcome variable = % of behavioral targets mastered SE: standard error; VIF: variance inflation factor; SE: standard error

Predictor Variables	Unstandardized Coefficients		Standardized Coefficients	t-value	p-value	95% Confidence Interval for B		Collinearity Statistics	
	B	SE	β			Lower Bound	Upper Bound	Tolerance	VIF
(Constant)	38.568	5.877		6.562	<0.001	26.89	50.246		
Age	0.329	0.329	0.1	1.001	0.32	-0.325	0.984	0.986	1.014
Average trials to mastery	-0.076	0.054	-0.243	-1.408	0.163	-0.183	0.031	0.329	3.043
Average teaching days to mastery	1.322	0.456	0.501	2.896	0.005	0.415	2.229	0.329	3.042

Causal moderation model: Model 2

Multiple regression causal moderation model summary indicates, for the dependent variable percentage of targets mastered and the predictor variables age, average trials to mastery, and average teaching days to mastery, R=0.448, R²=0.201, adjusted R²=0.174, R² change=0.201, F-change=7.448, df=3,89, and p=0.007 (the model is statistically significant). The model explains 20.1% of the variance in the percentage of targets mastered.

As indicated in Table 3 below, the dependent variable is the percentage of targets mastered, while the predictor variables are age, average trials to mastery, and average teaching days to mastery. As for coefficients, the constant is B=55.214, which is the baseline value of the percentage of targets mastered when all predictors are zero. The B for z-score age is -3.337, which is the unstandardized coefficient β=-0.126. Age alone has a negative but non-significant effect (p=0.300) on mastery. Each unit increase in age (standardized) results in a 3.337 decrease in the percentage mastered, but this is not statistically significant. The B for z-score average teaching days to mastery is 8.496, which is the unstandardized coefficient, β=0.318. Teaching days have a significant positive effect (p=0.001). Each unit increase in teaching days (standardized) leads to an 8.496 increase in the percentage mastered. The B for the age x teaching days interaction is -9.508, which is the unstandardized coefficient β=-0.391. The interaction between age and teaching days is significant (p=0.002). The negative coefficient indicates that as both age and teaching days increase together, the percentage of targets mastered decreases by 9.508 units. In terms of the collinearity statistics, tolerance and VIF, all values indicate no significant multicollinearity concerns. In terms of main effects, teaching days positively impact mastery significantly. Age alone does not significantly impact mastery. In terms of interaction effect, the interaction suggests that the positive effect of teaching days is reduced when combined with increased age. The moderation indicates that age affects how teaching days influence mastery. This model highlights the complexity of how age and teaching days together impact learning outcomes, emphasizing the importance of considering interactions in predictive models.

**Table 3:** Causal moderation model: Model #2 **Correlation is significant at p<.05, 2-tailed The data are presented as unstandardized coefficients (B), standard error, standardized coefficients (β), t-values, p-values, 95% confidence interval for B (upper and lower bounds), and collinearity statistics (tolerance and VIF) Outcome variable = % of behavioral targets mastered SE: standard error; VIF: variance inflation factor; SE: standard error

Predictor Variables	Unstandardized Coefficients		Standardized Coefficients	t-value	p-value	Collinearity Statistics	
	B	SE	β			Tolerance	VIF
(Constant)	55.214	2.505		22.041	<0.001		
Z-score: age	-3.337	3.204	-0.126	-1.041	0.3	0.617	1.621
Z-score: average teaching days to mastery	8.496	2.536	0.318	3.35	0.001	0.996	1.004
Age x teaching days interaction	-9.508	2.937	-0.391	-3.237	0.002	0.617	1.62

Implications

Moderating effect: The number of teaching days moderates the effect of age on mastery. The relationship between age and mastery is influenced by how many days it takes to teach.

Practical meaning: For older individuals or those requiring more teaching days to master, the percentage of targets mastered decreases significantly, suggesting that the combined effect of age and teaching days is important in understanding mastery outcomes.

The model suggests that while age alone does not significantly affect mastery, the interaction between age and teaching days does. More teaching days positively influence mastery, but this effect is moderated by age, with the interaction showing a significant decrease in mastery as both age and teaching days increase. This indicates that the interplay between age and teaching days has a substantial impact on the outcome, more so than each factor individually.

Causal moderation model: Model 3

The multiple regression causal moderation model summary indicates for the dependent variable, percentage of targets mastered, and the predictor variables, age, average trials to mastery, and average teaching days to mastery, R=0.371, R²=0.138, adjusted R²=0.109, R² change=0.138, F-change=4.745, df=3,89, and p=0.004 (the model is statistically significant). The model explains 13.8% of the variance in the percentage of targets mastered.

As indicated in Table 4 below, the dependent variable is the percentage of targets mastered, while the predictor variables are age, average trials to mastery, and average teaching days to mastery. As for coefficients, the constant is B=54.557, which is the baseline value of the percentage of targets mastered when all predictors are zero. The B for z-score age is 12.703, which is the unstandardized coefficient, β=0.478. Age alone has a negative and significant effect (p=0.002) on mastery. For each unit increase in the standardized age, the percentage of targets mastered increases by 12.703 units, with a VIF of 2.271. There is some multicollinearity present but generally acceptable. The B for z-score average trials to mastery is 3.130, which is the unstandardized coefficient, β=0.118. The average trials to mastery have a non-significant positive effect (p=0.241). For each unit increase in standardized trials to mastery, the percentage of targets mastered increases by 3.130 units, VIF = 1.027, which indicates low multicollinearity. The B for the age x average trials to mastery interactions is -1.139, which is the unstandardized coefficient, β=-0.466. The interaction between age and average trials to mastery is significant (p=0.002). The negative coefficient indicates that as both age and teaching days increase together, the percentage of targets mastered decreases by 1.139 units, VIF=2.309. There is some multicollinearity but acceptable. Interpretationally, age positively influences the percentage of targets mastered. The trials to mastery have a weak, non-significant positive effect. The interaction between age and trials suggests that as age increases, the positive effect of trials to mastery decreases significantly on the percentage mastered.

**Table 4:** Causal moderation model: Model #3 **Correlation is significant at p<.05, 2-tailed The data are presented as unstandardized coefficients (B), standard error, standardized coefficients (β), t-values, p-values, 95% confidence interval for B (upper and lower bounds), and collinearity statistics (tolerance and VIF) Outcome variable = % of behavioral targets mastered SE: standard error; VIF: variance inflation factor; SE: standard error

Predictor Variables	Unstandardized Coefficients		Standardized Coefficients	t-value	p-value	Collinearity Statistics	
	B	SE	β			Tolerance	VIF
(Constant)	54.557	2.603		20.958	<0.001		
Z-score: age	12.703	3.938	0.478	3.225	0.002	0.44	2.271
Z-score: average trials to mastery	3.13	2.65	0.118	1.181	0.241	0.974	1.027
Age x trials to mastery interaction	-0.139	0.044	-0.466	-3.119	0.002	0.433	2.309

This model indicates that age is a significant predictor, but its effect is moderated by the number of trials to mastery. Trials to mastery moderates the effect of age on target mastery. As one changes, the impact of the other also changes. For older individuals or those with more trials needed for mastery, the percentage of targets mastered tends to decrease, indicating a possible interaction effect that should be considered in interventions or strategies. The interaction term's significance highlights a complex relationship between these variables.

## Discussion

This study aimed to examine the potential associations between age and treatment intensity and their role in impacting significant behavioral outcomes as a result of ABA treatments. Specifically, this study sought to discern how these variables might interact to moderate the outcomes of ABA interventions. The primary metric for these outcomes was the proportion of behavioral objectives that were successfully mastered as a direct consequence of the ABA treatments. This exploration may be pivotal in understanding the nuanced dynamics of age and treatment intensity in shaping the effectiveness of ABA interventions. It might provide a foundation for future research and practical applications in behavioral therapy.

The results of this particular causal moderation analysis involved two models: Model 2 and Model 3. Model 1 embodied a fundamental multiple regression analysis, serving as the baseline for this investigation. This baseline model provides a foundational understanding of the relationships between predictor variables: age, average teaching days to mastery, and average trials to mastery, which was further refined and expanded upon in the two causal moderation models. Each model represents a different statistical relationship between variables, and their performance is evaluated based on several statistical measures.

Model 1 is the simplest model, with "average teaching days to mastery" as the significant predictor of the "% of targets mastered." However, it shows the weakest fit (R=0.355) among the three models, suggesting that this single predictor might not explain the variance in the outcome variable, "% of behavioral targets mastered."

Model 2 shows slight improvements over Model 1. It includes the main effect of "age" and an interaction effect of "age x average trials to mastery." The R-value (0.371) is higher than that in Model 1, indicating a stronger correlation. The standard error (25.06743) is lower than that in Model 1, suggesting more precise predictions. This model shows that age is a significant predictor, but its effect is moderated by the number of trials to mastery. The interaction term's significance highlights a complex relationship between these variables.

Model 3 is the best fit of the three. It includes the main effect of "average teaching days to mastery" and the interaction effect of "age x average teaching days to mastery." It has the highest R-value (0.448), indicating the strongest correlation. It also has the highest R^2^ (0.201) and adjusted R^2^ (0.174), suggesting it explains more variance in the dependent variable. Additionally, it has the lowest standard error of the estimate (24.13750), indicating the most precise predictions. The interaction suggests that the positive effect of teaching days is reduced when combined with increased age. The moderation shows that age affects how teaching days influence mastery. Its significance level (p<0.001) provides the most substantial evidence against the null hypothesis, suggesting a very low probability that the observed effects are due to chance.

Model 3, with its higher R-values and lower error, suggests a better overall fit and predictive power. This means it is more effective at predicting the "% of targets mastered" based on the given predictors. This could be crucial in understanding and improving the effectiveness of teaching methods in the study context.

Our results are similar to those of Virues-Ortega et al., whose study used multilevel models to analyze how different factors affect the success of an intervention. They found that the total time spent on the intervention (in hours) was the most critical factor in predicting positive outcomes, namely, the treatment's intensity (hours per week) and duration (total weeks) are crucial. More time spent on the intervention leads to better results, regardless of the participant's initial condition or age [16]. This was true even when they considered other factors, such as the participant's condition, before the intervention. We found r=0.826 (95% CI=0.752, 0.880), p<0.001, n=100 between average teaching days to mastery and % of behavioral targets mastered. Virues-Ortega et al. identified an age effect, which we did not observe. However, unlike our study, they did not investigate the interaction between age and the total time spent on the intervention [16].

Our findings align with those of Eldevick et al., who demonstrated through multiple regression models that their models explained a statistically significant, albeit small, portion of the variance in both IQ change (F(4, 211)=5.22, p<0.001, R²=0.090, adjusted R²=0.073) and ABC change (F(4,213)=14.45, p<0.001, R²=0.213, adjusted R²=0.199). Their analyses revealed that high intervention intensity was the sole variable that independently and positively predicted gains in both IQ and ABC. Additionally, initial ABC and IQ scores predicted ABC gains, with lower initial ABC scores correlating with larger ABC changes over two years, and higher initial IQ scores predicting greater ABC gains. Unlike our study, their study did not find any interaction terms to be significant independent predictors of changes in IQ or ABC [17].

Our findings are similar to those of Linstead et al., who conducted separate multiple linear regression analyses on 1468 children with ASD, aged 18 months to 12 years (M=7.57 ± 2.37 years), receiving individualized ABA services. Their results showed that both treatment intensity and duration were significant predictors of mastered learning objectives across all eight treatment domains. The academic and language domains had the strongest responses, with effect sizes of 1.68 and 1.85 for treatment intensity, and 4.70 and 9.02 for treatment duration, respectively. These findings align with previous research indicating that the total dosage of treatment positively influences outcomes. Their study provided a deeper understanding of how these treatment variables differentially impact various treatment domains [18]. However, unlike our study, they did not use moderation analysis or assess interactions.

Our results are consistent with those of Eckes et al., who conducted a moderator analysis of meta-analyzed studies. They identified potential interactions between treatment duration, intensity, total time spent in comprehensive ABA-based interventions, and age. Their evaluation using Q statistics revealed an interaction between age and treatment intensity for adaptive behavior, indicating that the impact of treatment intensity on post-treatment adaptive behavior diminishes with increasing age (β=-0.01, (-0.01, -0.00), QM(3)=74.45, p<0.001). However, the validity of these findings is limited due to the small number of eligible studies, suggesting that the results should be interpreted with caution. They do not mention the number of eligible studies [13].

While this study provides valuable insights into the potential associations between age, treatment intensity, and behavioral outcomes in ABA interventions, several limitations must be acknowledged. The sample size used in this study may limit the generalizability of the findings. A larger and more diverse sample would be necessary to confirm the robustness of the results and to ensure that they are applicable to a broader population. Also, there were seven missing values for the variable "age," which were the result of seven instances where the individuals' ages were not recorded due to random data entry errors (MCAR).

The study relies on specific metrics such as "average teaching days to mastery" and "average trials to mastery" to gauge treatment intensity. These measures, while useful, may not capture the full complexity of treatment intensity and its impact on behavioral outcomes. Future research could benefit from incorporating additional or alternative measures of treatment intensity. The cross-sectional nature of the study limits the ability to draw causal inferences. Longitudinal studies would be more effective in establishing causal relationships between age, treatment intensity, and behavioral outcomes over time.

Although the study attempts to control for various factors, there may be other confounding variables that were not accounted for, such as the severity of behavioral issues, the specific nature of the ABA interventions, and individual differences in response to treatment. These unmeasured variables could influence the results. The use of multiple regression and causal moderation models, while sophisticated, may have inherent limitations. The models assume linear relationships between variables, which may not fully capture the complexity of the interactions. Additionally, the models' fit, as indicated by R-values and standard errors, suggests that there is still unexplained variance in the outcome variable. The study's focus on a specific population undergoing ABA treatment may limit the applicability of the findings to other populations or types of behavioral interventions. Further research is needed to explore whether these findings hold true in different contexts and with different intervention strategies. By addressing these limitations in future research, researchers can build a more comprehensive understanding of how age and treatment intensity interact to influence the effectiveness of ABA interventions, ultimately leading to more tailored and effective therapeutic approaches.

## Conclusions

This study provides insights into the complex interplay between age, treatment intensity, and behavioral outcomes of ABA interventions. By employing causal moderation analysis across two models, we sought to illuminate how these factors contribute to the effectiveness of ABA treatments, specifically in terms of the mastery of behavioral objectives. Model 1, while serving as a foundational multiple regression analysis, highlighted that average teaching days significantly predict the percentage of targets mastered. However, the model's limited explanatory power suggests that additional variables influence these outcomes. Models 2 and 3 advanced this understanding by incorporating interaction effects, demonstrating that age moderates the relationship between treatment variables and behavioral outcomes. Model 3 emerged as the most robust, with its higher R-values and lower standard error, underscoring the nuanced role age plays in modifying the impact of teaching days on mastery.

For practitioners, understanding the moderating effect of age can refine intervention strategies, ensuring they are tailored to maximize efficacy across different age groups. For researchers, this study lays the groundwork for further exploration into other potential moderators, such as cognitive or environmental factors, that might influence ABA outcomes. This study enhances our comprehension of the dynamic interactions between age and treatment intensity within ABA interventions. By recognizing and leveraging these interactions, practitioners and researchers can optimize therapeutic strategies, ultimately fostering more effective and personalized approaches to behavioral therapy.

## References

[1] (2024). Early identification of autism spectrum disorder among children aged 4 years — autism and developmental disabilities monitoring network, 11 sites, United States, 2020. https://www.cdc.gov/mmwr/volumes/72/ss/ss7201a1.htm.

[2] Peterson T, Dodson J, Sherwin R, Strale F (2024). Comparative effects of applied behavior analysis on male and female individuals with autism spectrum disorder. Cureus.

[3] American Psychiatric Association (2022). The Diagnostic and Statistical Manual of Mental Disorders, Fifth Edition.

[4] Peterson T, Dodson J, Strale F (2024). Replicative study of the impacts of applied behavior analysis on target behaviors in individuals with autism using repeated measures. Cureus.

[5] Peterson T, Dodson J, Hisey A, Sherwin R, Strale F (2024). Examining the effects of discrete trials, mass trials, and naturalistic environment training on autistic individuals using repeated measures. Cureus.

[6] Peterson T, Dodson J, Strale F (2024). Impact of applied behavior analysis on autistic children target behaviors: a replication using repeated measures. Cureus.

[7] Peterson T, Dodson J, Strale F (2024). Treating target behaviors of autistic individuals with applied behavior analysis: an ongoing replication study. Cureus.

[8] (2024). Treatment and intervention for autism spectrum disorder. https://www.cdc.gov/autism/treatment/index.html.

[9] Yu Q, Li E, Li L, Liang W (2020). Efficacy of interventions based on applied behavior analysis for autism spectrum disorder: a meta-analysis. Psychiatry Investig.

[10] Makrygianni M, Gena A, Katoudi S, Galanis P (2018). The effectiveness of applied behavior analytic interventions for children with autism spectrum disorder: a meta-analytic study. Res Autism Spectr Disord.

[11] Dixon M, Paliliunas D, Barron B, Schmick A, Stanley C (2021). Randomized controlled trial evaluation of ABA content on IQ gains in children with autism. J Behav Educ.

[12] Rodgers M, Marshall D, Simmonds M (2020). Interventions based on early intensive applied behaviour analysis for autistic children: a systematic review and cost-effectiveness analysis. Health Technol Assess.

[13] Eckes T, Buhlmann U, Holling HD, Möllmann A (2023). Comprehensive ABA-based interventions in the treatment of children with autism spectrum disorder - a meta-analysis. BMC Psychiatry.

[14] (2024). ‘Autism can be reversed’, scientists claim. https://www.telegraph.co.uk/news/2024/07/20/severe-autism-can-be-reversed-groundbreaking-study-suggests/.

[15] Granpeesheh D, Tarbox J, Najdowski A, Kornak J (2014). Evidence-based Treatment for Children With Autism: The CARD Model.

[16] Virues-Ortega J, Rodríguez V, Yu C (2013). Prediction of treatment outcomes and longitudinal analysis in children with autism undergoing intensive behavioral intervention. Int J Clin Health Psychol.

[17] Eldevik S, Hastings RP, Hughes JC, Jahr E, Eikeseth S, Cross S (2010). Using participant data to extend the evidence base for intensive behavioral intervention for children with autism. Am J Intellect Dev Disabil.

[18] Linstead E, Dixon DR, Hong E, Burns CO, French R, Novack MN, Granpeesheh D (2017). An evaluation of the effects of intensity and duration on outcomes across treatment domains for children with autism spectrum disorder. Transl Psychiatry.

[19] Lovaas OI (1987). Behavioral treatment and normal educational and intellectual functioning in young autistic children. J Consult Clin Psychol.

[20] Reichow B, Wolery M (2009). Comprehensive synthesis of early intensive behavioral interventions for young children with autism based on the UCLA young autism project model. J Autism Dev Disord.

[21] Smith T, Eikeseth S, Klevstrand M, Lovaas O (1997). Intensive behavioral treatment for preschoolers with severe mental retardation and pervasive developmental disorder. Am J Ment Retard.

[22] Peterson T, Dodson J, Strale F (2024). Predicting behavioral target mastery with age, intensity and duration, open targets, and maintenance failure with applied behavior analysis in individuals with autism. Cureus.

[23] Kromrey J, Foster-Johnson L (1998). Mean centering in moderated multiple regression: much ado about nothing. Educ Psychol Meas.

[24] Peterson T, Dodson J, Sherwin R, Strale F (2024). An internal consistency reliability study of the Catalyst Datafinch applied behavior analysis data collection application with autistic individuals. Cureus.

[25] Catalyst by DataFinch. (2022). Catalyst was created by ABA providers, for ABA providers. [ Apr; 2024 ]. 2022 (2024). Catalyst. https://datafinch.com/about/.

[26] IBM SPSS Statistics 29. [ Dec; 2023 ]. 2023 (2024). Downloading IBM SPSS statistics 29. https://www.ibm.com/support/pages/downloading-ibm-spss-statistics-29.

